# Efficacy of Systemically Administered Retargeted Oncolytic Herpes Simplex Viruses—Clearance and Biodistribution in Naïve and HSV-Preimmune Mice

**DOI:** 10.3390/cancers15164042

**Published:** 2023-08-10

**Authors:** Andrea Vannini, Federico Parenti, Catia Barboni, Cristina Forghieri, Valerio Leoni, Mara Sanapo, Daniela Bressanin, Anna Zaghini, Gabriella Campadelli-Fiume, Tatiana Gianni

**Affiliations:** 1 Department of Medical and Surgical Sciences, University of Bologna, 40126 Bologna, Italy; andrea.vannini5@unibo.it (A.V.); federico.parenti5@unibo.it (F.P.); cristina.forghieri@unibo.it (C.F.);; 2 Department of Pharmacy and Biotechnology, University of Bologna, 40126 Bologna, Italy; 3 Department of Veterinary Medical Sciences, University of Bologna, 40126 Bologna, Italy; catia.barboni@unibo.it (C.B.); anna.zaghini@unibo.it (A.Z.); 4 Animal Facility Unit, Biogem, 83031 Ariano Irpino, Italy; mara.sanapo@biogem.it

**Keywords:** oncolytic virus, oncolytic herpes simplex virus, retargeting, HER2, PSMA, anti-cancer vaccine, systemic oncolytic therapy, oncolytic virus biodistribution, oncolytic virus immunity

## Abstract

**Simple Summary:**

Oncolytic viruses were introduced as innovative anticancer agents in preclinical and clinical settings. Upon intralesional administration, they directly kill the cancer cells and also stimulate the anticancer immunity, i.e., function as immunotherapeutics. Thus, owing oncolytic and immunostimulatory activities, they inhibit the growth not only of the injected tumors but also, in part, of distant untreated lesions. Although systemic administration of oncolytic viruses would be the ideal treatment for hard-to-reach and metastatic cancers, blood antiviral factors—especially prior immunity to the virus -, tissue absorption, and off-tumor infections hinder this route of administration. The tropism-retargeted oncolytic herpes viruses (ReHVs) were designed to avoid the off-tumor infections consequent to the widespread occurrence of the viral natural receptors. ReHVs specifically infect those cancer cells that express a tumor-specific receptor of choice and spare cells harboring the natural viral receptors. Given their ability to not cause off-tumor and off-target infections, ReHVs are more suitable for systemic delivery.

**Abstract:**

We investigated the anticancer efficacy, blood clearance, and tissue biodistribution of systemically administered retargeted oncolytic herpes simplex viruses (ReHVs) in HSV-naïve and HSV-preimmunized (HSV-IMM) mice. Efficacy was tested against lung tumors formed upon intravenous administration of cancer cells, a model of metastatic disease, and against subcutaneous distant tumors. In naïve mice, HER2- and hPSMA-retargeted viruses, both armed with mIL-12, were highly effective, even when administered to mice with well-developed tumors. Efficacy was higher for combination regimens with immune checkpoint inhibitors. A significant amount of infectious virus persisted in the blood for at least 1 h. Viral genomes, or fragments thereof, persisted in the blood and tissues for days. Remarkably, the only sites of viral replication were the lungs of tumor-positive mice and the subcutaneous tumors. No replication was detected in other tissues, strengthening the evidence of the high cancer specificity of ReHVs, a property that renders ReHVs suitable for systemic administration. In HSV-IMM mice, ReHVs administered at late times failed to exert anticancer efficacy, and the circulating virus was rapidly inactivated. Serum stability and in vivo whole blood stability assays highlighted neutralizing antibodies as the main factor in virus inactivation. Efforts to deplete mice of the neutralizing antibodies are ongoing.

## 1. Introduction

Oncolytic viruses (OVs) were introduced as innovative tools in anticancer therapy some twenty years ago at the preclinical level [1,2,3]. It was later recognized that their efficacy rested in part in their ability to elicit an anticancer immune response; hence they were renamed onco-immunotherapeutic viruses (OIVs) and are now recognized as a branch of cancer immunotherapy [4]. OIVs have been derived from various viral families, each with peculiar pros and cons [5,6]. The OVs that so far have received approval for clinical use include an echovirus, an adenovirus, and two herpes simplex virus (HSV) derivatives. An echovirus was first approved in Latvia and subsequently in the U.S. by FDA as an orphan drug [7]. The Oncorine Adenovirus was approved in 2005 in China for nasopharyngeal carcinoma, and the World First commercialized Oncolytic Virus Medicine [8]. T-VEC, also named OncoVEX^GM-CSF^ (Imlygic), was approved in US and EU against cutaneous melanoma [9], and Delytact was recently approved in Japan against glioblastoma multiforme [10].

The approved oncolytic HSVs (oHSVs) are administered intratumorally (i.t.). Their ability to contrast relapsing and metastatic disease rests on innate and adaptive immune responses and subsequent immunotherapeutic effects. Ideally, to contrast the hard-to-reach and metastatic cancers, one would like to administer OIVs systemically and let the viruses search for, populate, and kill the tumors [11,12,13,14,15,16,17,18,19]. The main obstacle to the systemic delivery of OVs lies primarily in the prior immunity that exists in the human population against the employed OVs, which results in the abolishment or reduction of the anticancer efficacy and is affected by the proximity between the site of virus administration and target tumor [20,21,22]. To circumvent this obstacle, numerous strategies have been put in place. One consisted of the development of OVs from animal viruses. However, repeated administrations elicit an immune response, and eventually, the problem of antiviral immunity is not fully overcome [23]. Alternatively, viruses have been delivered by mesenchymal stem cells. Thus they have been made invisible to the immune system [24,25]. Secondly, the receptors that OVs use to enter cells are widespread in human tissues, a condition that favors possible off-tumor and off-target infections—whether productive or nonproductive—and affects the overall safety profile. At the preclinical level, anticancer efficacy in virus-immune mice has been demonstrated in some models [20,21,22,26]. Clinical trials to test the efficacy of systemically administered OIVs are ongoing, with still unsatisfactory results [13,27,28].

Our laboratory designed and preclinically tested a distinct type of OI Herpesviruses, retargeted to a cancer-specific receptor of choice, referred to as retargeted Herpesviruses (ReHVs). In contrast to the wild-type HSVs that infect cells through their natural receptors nectin1 and HVEM (Herpes virus entry mediator), ReHVs carry on their surface a novel ligand that enables infection of cells carrying the ligand-receptor [29,30,31]. The ligand is inserted into one of the entry glycoproteins, gD, gH, or gB [32,33,34,35]. The ligand receptor is chosen among the tumor-associated antigens (TAAs). This strategy results in herpesviruses that specifically infect cancer cells and fail to infect cells and tissues usually targeted by wt-HSV. In addition, TAAs are genetically amplified and overexpressed in cancer tissues, a property that contributes to the specific tropism of ReHVs. Despite this, the cancer specificity is not absolute, and any selected receptor, although not widespread in human tissues, can still be present in one or another tissue. Essentially, ReHVs differ from the previous oHSVs. The latter are pan-tumor agents, whereas ReHVs fall into the precision medicine approach. Each ReHV targets the set of indications that share the same TAA. So far, this laboratory has generated ReHVs readdressed to HER2, PSMA, EGFR, EGFRvIII, mesothelin, and nectin4 [30,33,36,37,38,39]. Their specificity, safety profile, and anticancer efficacy upon i.t. administration have been extensively documented [30,39,40,41]. Given their ability to not cause off-tumor and off-target infections, in principle ReHVs are more suitable for systemic delivery than pantropic oHSVs. Preliminary evidence has been provided for their systemic efficacy against lung tumors formed upon intravenous (IV) injection of tumor cells [22], a model of metastatic disease.

The objectives of this study were to demonstrate the efficacy of systemically delivered ReHVs in HSV-naïve or previously immunized (HSV-IMM) mice against lung tumors syngeneic with the BALB/c mice and subcutaneous tumors syngeneic with C57BL/6 mice, to carry out clearance and biodistribution analyses in HSV-naive and HSV-IMM mice, and to provide insights into the blood factors that limit the efficacy of systemic administration.

## 2. Materials and Methods

Cells and viruses. SK-OV-3 human ovarian cancer cells (Roswell Park Memorial Institute), CT26 murine colon carcinoma cells (ATCC), and their derivatives were cultured in RPMI-Glutamax (Thermo Fisher, Waltham, MA, USA) containing 10% fetal bovine serum (FBS). Mouse Lewis lung carcinoma LLC1 (ATCC) cells and their derivatives were cultured in Dulbecco’s Modified Eagle’s Medium (Thermo Fisher). R-337 and R-405 were described [30,37] and cultured in SK-OV-3 and SK-OV-3-hPSMA cells, respectively. The R-8102 virus was described [42].

In vivo experiments. BALB/c mice transgenic for and tolerant to human HER2 (BALB/c-TG) were previously described [40], and C57BL/6 mice were obtained from Charles River Laboratories (Wilmington, MA, USA); both mouse strains were bred in the facility of the Department of Veterinary Medical Sciences, University of Bologna. Male and female mice were used randomly for the experiments, in accordance with the request from the Ethics Committee. For intravenous (IV) therapy of metastatic tumors, CT26-HER2 cells were injected IV into the tail vein of 12-week-old BALB/c-TG mice in 100 μL of serum-free medium. After the indicated days, R-337 was administered IV 1 to 4 times at the indicated doses (1 × 10^7^–1 × 10^8^ PFUs/injection) in 100 μL of PBS or vehicle (see figure legends for details). In the early treatment scheme, 1 × 10^6^ CT26-HER2 cells were used to establish lung metastases, and mice were treated IV with R-337 at days 2–3 after cell injection and sacrificed at day 17. In the late treatment scheme, 3 × 10^5^ CT26-HER2 cells were used to establish lung metastases, and mice were treated IV with R-337 at day 10 after cell injection and sacrificed at day 21. Where indicated, mice received 3 i.p. injections of anti-CTLA-4 antibody (100 µg of antibody in 100 µL of PBS per injection, clone 9H10; BioXcell, Lebanon, NH, USA) or vehicle (100 µL of PBS) at 3–4-day intervals. At the time of sacrifice, lungs were harvested, perfused with the staining solution (15% India ink in PBS), and fixed in Fekete’s solution (61% ethanol, 32% water, 4% acetic acid, and 3% formaldehyde). Lung metastatic colonies were counted under the microscope as white spots. For intravenous therapy in the subcutaneous tumor setting, 1 × 10^6^ LLC1-hPSMA cells were implanted subcutaneously in the left flank of 12-week-old C57BL/6 mice in 100 μL of serum-free medium. Tumor volumes were measured 2 times a week by measuring the major and minor diameters and calculating the volumes by the formula: major diameter × (minor diameter)^2^ × 0.5. Mice were sacrificed when their tumors reached a volume of about 1500 mm^3^, ulceration occurred, or the animals showed distress or pain. Nine days after tumor grafting, when tumor volume averaged 70–100 mm^3^, mice received 2 IV injections of R-405 (1 × 10^8^ PFU for each injection in 100 µL of PBS) or vehicle at an interval of 6 days. Where indicated, mice received 3 i.p. injections of mouse anti-PD-1 antibody (100 µg of antibody in 100 µL PBS per injection, clone RMP1-14, BioXcell), or vehicle, at 5-day intervals. For the experiments in HSV-IMM animals, mice were immunized twice with a mixture of untreated and UV-inactivated HSV-1(F) virions [43] (1 × 10^5^ and 2 × 10^5^ PFU per injection, respectively), 6 and 3 weeks before injection of tumor cells or R-337. For biodistribution experiments, 1 × 10^6^ CT26-HER2 cells were injected IV into the tail vein of BALB/c-TG mice (naïve or HSV-1-immune animals). After 7 or 12 days, mice received an IV injection of R-337 (1 × 10^7^ PFUs in 100 µL of PBS). Where indicated, R-337 was IV injected in tumor-negative mice. Mice were sacrificed at the indicated times, and blood and tissue were collected.

R-337 biodistribution and replication in mouse tissues. To determine the biodistribution of R-337 in blood and tissues after IV injections, a few mg of tissue homogenates or 20 μL of blood were used for DNA purification with the Nucleospin Tissue kit (Macherey-Nagel, Düren, Germany) according to the manufacturer’s protocol. HSV genome copies (g.c.) were quantified by qRT-PCR: 50 ng of DNA extracted from tissues or the amount of DNA obtained from 1 μL of blood were mixed with 5 μL of TaqMan Fast Advanced Master Mix (Applied Biosystems, Waltham, MA, USA) and 0.5 μL of HSV DNApol primer/probe [39] in a final volume of 10 μL. The qRT-PCR reactions were performed in a StepOnePlus system (Applied Biosystems), following the protocol indicated in the Master Mix. The amount of g.c. was determined by comparison with a standard curve, prepared using purified genomic HSV DNA, and expressed as g.c./100 ng of DNA or g.c./100 μL blood [39]. To detect human HER2 in tissue homogenates, 50 ng of purified DNA was assayed by qRT-PCR with a Taqman primer/probe for human HER2 (HER2-nect_RTF AGAGCCAGCCCTGTTAACTC, HER2-nect_RTR CTGGCTGCACTTCCCAGA, HER2-nect_probe [FAM]-CTAACATCGCGGCCGCGCCG-[TAMRA]). To determine the kinetics of R-337 clearance from blood, 20 μL of blood samples were taken from mice, immediately mixed with 2.5 mM EDTA to prevent clotting, serially diluted, and plated on SK-OV-3 cells. After 90 min incubation, the infected cultures were overlaid with an agar-containing medium, and the number of plaques was scored 5 days later. To determine R-337 replication in tissues, a few mg of tissue homogenates were employed for RNA purification by means of the NucleoSpin RNA kit (Macherey-Nagel), including in-column DNaseI treatment. For RT-PCR assays, 2 μg of total RNA was used for cDNA synthesis using the high-capacity cDNA Reverse Transcription Kit (Applied Biosystems) in a 20 μL reaction. The qRT-PCR reactions were performed in a StepOnePlus system (Applied Biosystems) using 0.5 μL of cDNA for each assay. The TaqMan primers/probes used for the assay were HSV-1 glycoprotein C (gC_taqf ACCTTCACCTGCCAGATGAC, gC_taqr ACATGCCGGACCAAATTC, gC_taq_probe [FAM]-CCAGGGCCAGCGGTGGCA-[TAMRA]) and mm01612987_g1 (Rpl13a). Expression levels were determined by the ΔΔCt method, normalized to Rpl13a.

Partial depletion of immune factors. Mice immunized to HSV-1 or naïve were treated i.p. with Cobra Venom Factor (DBA; 5 U and 1.6 U in 100 µL PBS, 2 days and 1 h before R-337 injection, respectively) and cyclophosphamide (Merck, Darmstadt, Germany; 2.5 mg in 250 µL PBS) 2 days before R-337 injection. To temporarily reduce the neutralizing antibody to HSV-1, mice immunized to HSV-1 or naïve were injected IV with UV-inactivated HSV-1 (F) (2 × 10^8^ PFUs in 100 µL PBS) the day before R-337 injection. After R-337 administration, blood samples were taken after 2 or 30 min, mixed with 2.5 mM EDTA to prevent coagulation, serially diluted, and plated on SK-OV-3 cells. For the in vitro assay, 50 µL of sera from HSV-1-immunized or HSV-1-naïve mice were heated at 56 °C for 30 min for complement inactivation or treated with 50 μL Sepharose Protein G (Pierce Biotechnology, Waltham, MA, USA) on ice for 1 h for antibody depletion. 1 × 10^6^ PFUs of R-337 were added to treated and untreated sera and incubated for 30 min, then serially diluted and plated on SK-OV-3 cells for virus titer determination. The 100% value represents the data obtained with untreated sera from HSV-naïve mice.

Determination of splenocyte reactivity to cancer cells. Freshly explanted spleens were smashed through a 70 μm cell strainer, red blood cells in the spleens were lysed with ACK buffer (150 mM NH4Cl, 10 mM NaHCO_3_, 1 mM EDTA), and splenocytes were resuspended in culture medium (RPMI 1640 containing 10% heat-inactivated FBS, 1% penicillin/streptomycin), counted and seeded in 24-well plates. CT26-wt, CT26-HER2, LLC1-wt, or LLC1-hPSMA cell suspensions were treated with 15 μg/mL mitomycin (Merck) for 2 h at 37 °C, then washed 3 times with fresh medium. Splenocytes (1 × 10^6^ cells/well) were co-seeded with 1 × 10^5^ cells treated with mitomycin in 0.5 mL of medium and co-cultured for 72 h. The media were collected, and the amount of secreted IFNγ was quantified by ELISA (IFN-gamma Mouse ELISA Kit, Thermo Fisher).

Determination of serum antibodies to cancer cells and HSV. To determine antibodies against tumor cells, mouse sera diluted 1:150 in FACS buffer were incubated with 1.5 × 10^5^ CT26-wt, CT26-HER2, LLC1-wt, or LLC1-hPSMA cells in a 96-well plate for 1 h on ice. Cells were washed 3 times with cold FACS buffer, incubated with anti-mouse APC (1:200, eBioscience, San Diego, CA, USA) for 1 h on ice, and washed 3 times. Fluorescence was quantified with BD C6 Accuri. To determine the antibodies against HSV-1, a cell enzyme-linked immunosorbent assay (CELISA) was performed as described [30]. Briefly, RS cells were infected with HSV-1 (F) at 3 PFU/cell in a 96-well plate. Twenty-four hours later, cells were reacted with mouse sera diluted 1:60, with MAb HD1 [44] diluted 1:400, or with human sera from anonymous HSV-1-positive patients. Then cells were fixed with paraformaldehyde and reacted with anti-mouse peroxidase (mouse sera and HD1) or anti-human peroxidase (human sera). Finally, peroxidase substrate o-phenylenediamine hydrochloride (OPD; Merck) was added, and plates were read at 490 nm with GloMax Discover System (Promega Corporation, Madison, WI, USA). The neutralization potency of mouse sera on HSV-1 was determined as inhibitory concentration values (IC50) of sera to HSV-1 infection. R-8102 virions (3 PFU/cell) were pre-incubated with serially diluted heat-inactivated mice and human sera (30 min at 56 °C) or increasing amounts of MAb HD1, in 50 μL of medium for 1 h at 37 °C, and then allowed to adsorb to RS cells for 90 min. The viral inoculum was removed, and the cells were rinsed twice, covered with a medium containing the same concentration of antibodies or sera as in the pre-absorption step, shifted to 37 °C, and incubated for 16 h. β-Galactosidase activity was assayed as previously described [45], and the inhibition of infection was calculated as the ratio of serum-treated samples to control. For each serum or MAb HD1, the infection inhibition versus dilution curve was used to calculate IC50 values with GraphPad Prism 8 software.

## 3. Results

### 3.1. Therapeutic Efficacy of Systemically Administered R-337 Monotherapy against CT26-HER2 Lung Tumors, a Model of Metastatic Disease. Early Administration

**Figure 1:**
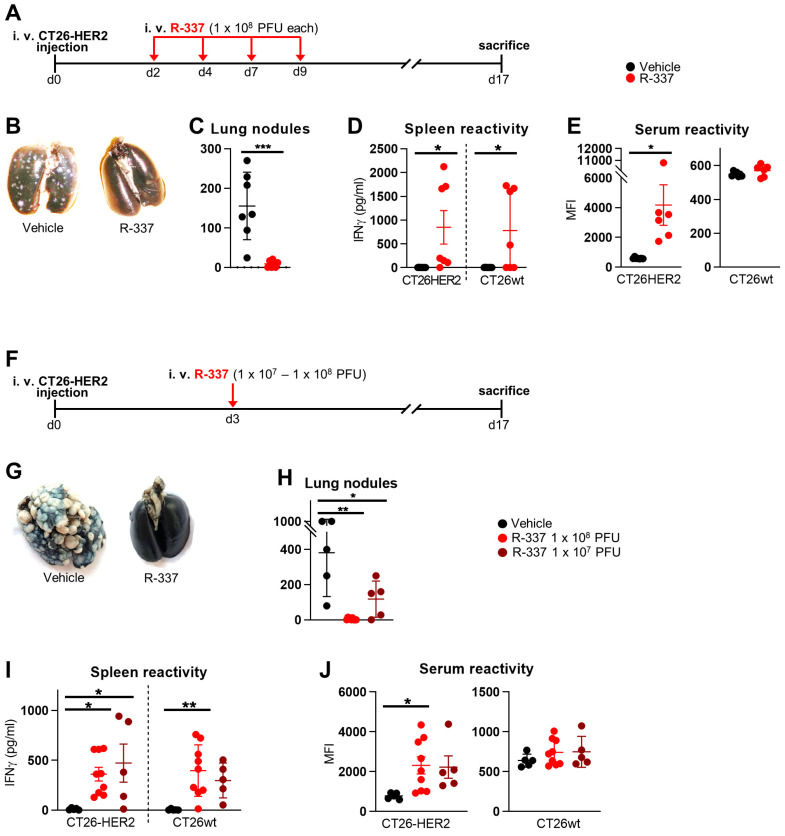
Efficacy of IV monotherapy with R-337 on the growth of metastatic CT26-HER2 tumors. (**A**) Early treatment scheme with multiple IV injections. BALB/c-TG mice were injected IV with 1 × 10^6^ CT26-HER2 cells to establish metastatic lung tumors and, 2 days later, received 4 IV injections of R-337 (1 × 10^8^ PFU per injection) every 2–3 days. Mice were sacrificed on day 17 after tumor cell injection. At the time of sacrifice, lungs were harvested, perfused with the staining solution, fixed in Fekete’s solution, and lung metastatic nodules were counted under a microscope. (**B**) Representative images of stained lungs from groups treated with vehicle and R-337. Normal lung tissue is black, while metastases are white spots. (**C**) The number of tumor nodules counted on the surface of lungs from vehicle- and R-337-treated mice. (**D**,**E**) Immune response to CT26-wt and CT26-HER2 tumor cells in splenocytes (**D**) and sera (**E**) taken at the time of sacrifice from vehicle- and R-337-treated groups. (**F**) Scalar doses of R-337 in the early treatment schedule. BALB/c-TG mice were injected IV with 1 × 10^6^ CT26-HER2 cells and, 3 days later, received 1 IV injections of R-337 (1 × 10^7^ or 1 × 10^8^ PFUs). Mice were sacrificed on day 17 after tumor cell injection. At the time of sacrifice, lungs were harvested and stained, and lung metastatic nodules were counted. (**G**) Representative images of the stained lungs of the vehicle-treated and R-337-treated groups. (**H**) The number of tumor nodules counted on the surface of the lungs. (**I**,**J**) Immune response to CT26-wt and CT26-HER2 tumor cells in splenocytes (**I**) and sera (**J**) collected at the time of sacrifice. Each circle corresponds to an individual mouse; the horizontal line indicates the mean value, and the vertical bars ± SD. (**C**–**E**,**H**–**J**) Statistical significance was calculated by t-test (**C**–**E**) or ANOVA test with Tukey’s correction (**H**–**J**) and expressed as * = *p*-value < 0.05; ** = *p*-value < 0.01; *** = *p*-value < 0.001. Color code: mice treated with vehicle in black, R-337 (1 × 10^8^ PFUs) in red, and R-337 (1 × 10^7^ PFUs) in burgundy.

To test the therapeutic efficacy of systemically delivered R-337 in a model of metastatic disease, mice were first engrafted with IV-administered CT26-HER2 cells. By this route, multiple tumors are localized to the lungs. CT26 tumors are syngeneic with BALB/c mice. To mimic the situation in humans more faithfully, we employed BALB/c-TG mice transgenic/tolerant to human HER2 that we had previously generated [40]. The mice were then treated IV with R-337, according to the schedule shown in Figure 1A, and sacrificed at d 17. Figure 1B,C show that repeated doses of R-337 monotherapy exerted almost complete protection. The mice exhibited a strong T cell response, seen as splenocyte reactivity to CT26-HER2 cells, supporting the view that the protection might, in part, be immune-mediated (Figure 1D). Remarkably, the mice developed a T cell response also against agnostic tumor antigens expressed by CT26-wt cells (Figure 1D). The antibodies response was directed primarily to HER2 (Figure 1E) and to a limited extent to the CT26-wt tumor cell antigens (trend, not statistically significant).

Next, we asked whether a single dose of R-337 in decreasing amounts was sufficient to inhibit tumor growth in the same therapeutic setting (Figure 1F). Figure 1G shows that a single 1 × 10^8^ PFU dose resulted in a complete cure. The 1 × 10^7^ PFUs dose reduced the number of tumor nodules in the lungs in a dose-dependent manner (Figure 1H). Thus, a single dose of R-337 monotherapy was sufficient for a complete response. The T and B cell responses were very similar to those exerted by the multiple doses, i.e., the T cell response was addressed to the HER2 antigen as well as to the agnostic tumor antigens (Figure 1I), whereas the B response was addressed mainly to HER2 (Figure 1J). Two immunotherapeutic features were worth of note in the two series of experiments. Inasmuch as the mice were transgenic/tolerant to human HER2, a situation that mirrors the one in patients carrying HER2-positive tumors, the treatment broke the tolerance to human HER2 and effectively vaccinated against HER2. Secondly, the treatment elicited a response not only to the immunodominant HER2 antigen but also to agnostic tumor antigens.

### 3.2. Combination with α-CTLA4 Antibodies Increases the Therapeutic Efficacy of Systemically Administered R-337

**Figure 2:**
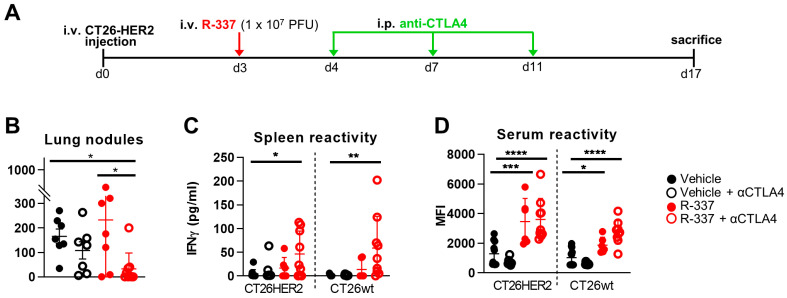
Efficacy of IV therapy with R-337 in combination with anti-CTLA-4 antibody (αCTLA4) on the growth of metastatic CT26-HER2 tumors. (**A**) Scheme of early treatment with a single virus injection. BALB/c-TG mice were injected IV with 1 × 10^6^ CT26-HER2 cells to establish metastatic lung tumors and, 3 days later, received 1 IV injection of R-337 (1 × 10^7^ PFU per injection) and 3 i.p. injections of anti-CTLA-4 MAb (αCTLA4) at 3–4-day intervals. Mice were sacrificed on day 17 after tumor cell injection. At the time of sacrifice, lungs were harvested, perfused with the staining solution, fixed in Fekete’s solution, and lung metastatic nodules were counted under a microscope. (**B**) The number of tumor nodules counted on the surface of the lungs. (**C**,**D**) Immune response to CT26-wt and CT26-HER2 tumor cells in splenocytes (**C**) and sera (**D**) collected at the time of sacrifice. Each circle corresponds to an individual mouse; the horizontal line indicates the mean value, and the vertical bars ± SD. (**B**–**D**) Statistical significance was calculated by ANOVA test with Tukey’s correction and expressed as * = *p*-value < 0.05; ** = *p*-value < 0.01; *** = *p*-value < 0.001; **** = *p*-value < 0.0001. Color codes: mice treated with vehicle (black) or R-337 (red). Open circles indicate combination therapy with αCTLA4 antibody, while filled circles indicate no αCTLA4 antibody.

Next, we ascertained whether the therapeutic efficacy of R-337 increased when the virus was administered in combination with α-CTLA4 antibodies. R-337 was injected as a single low dose (1 × 10^7^ PFU) that exerts incomplete reduction in tumor growth as monotherapy. Four groups of mice received the vehicle, α-CTLA4 antibody monotherapy, R-337 monotherapy, and the combination of R-337 and α-CTLA4 antibody. The schedule is shown in Figure 2A. Figure 2B shows that the antibody alone or R-337 alone did not significantly alter tumor growth. When combined, the two treatments significantly reduced the tumor burden relative to vehicle or R-337 monotherapy, implying an additional or synergistic effect and an overall advantage of the combination regimen, in agreement with previous findings from our laboratory on this tumor model [30,40]. The T and B cell responses were similar to those observed in the R-337 monotherapy experiments (Figure 2C), except that the combination therapy elicited an antibody response to both HER2 and wild-type CT26 cells (Figure 2D).

### 3.3. Systemically Administered ReHV R-405 Inhibits the Growth of a Subcutaneous Tumor

**Figure 3:**
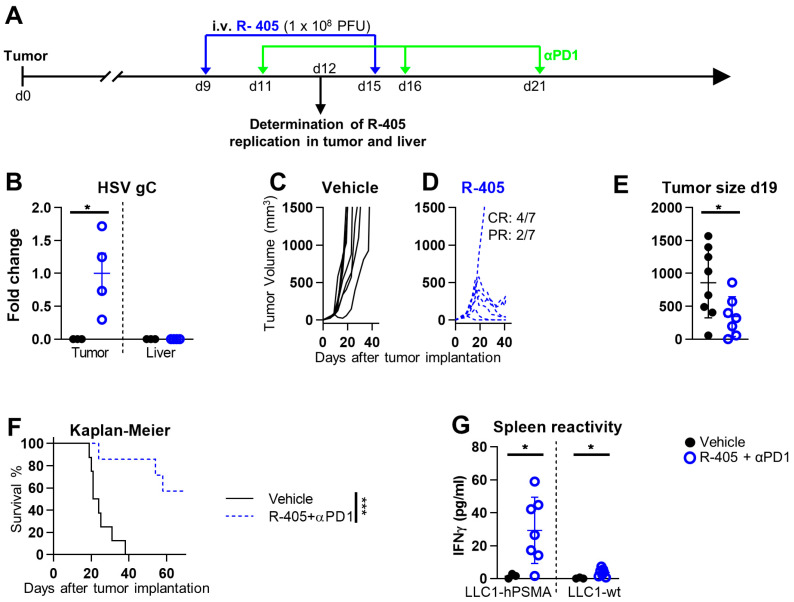
Efficacy of IV therapy with R-405 in combination with anti-PD-1 antibody (αPD1) on the growth of subcutaneous LLC1-hPSMA tumors. (**A**) Scheme of treatments. C57BL/6 mice were implanted s.c. in the left flank with 1 × 10^6^ LLC1-hPSMA cells. 9 days later, when the tumor volume averaged 70–100 mm^3^, mice received 2 IV injections of R-405 (1 × 10^8^ PFU) or vehicle at 6-day intervals and 3 i.p. injections of anti-PD-1 MAb (αPD1) at 3–4-day intervals. (**B**) Replication of HSV in tumors and livers of R-405 treated and untreated mice. mRNA levels of viral glycoprotein gC were determined by qRT-PCR in tumor and liver samples. Normalized data on endogenous control Rpl13a were reported by setting the mean value of gC expression in tumors in the R-405-treated group to 1. (**C**,**D**) Tumor growth curves. The numbers shown in the panel indicate the number of mice that were completely cured of tumor (complete response, CR) or showed delayed/reduced tumor growth (partial response, PR). Mice were classified as PR when the tumor volume was 50% smaller than the mean tumor size in the vehicle group in at least 2 consecutive measurements. (**E**) Primary tumor volumes at day 19 after implantation. (**F**) Kaplan-Meier survival curves of the 2 groups of mice. (**G**) Immune response to LLC1-wt and LLC1-hPSMA tumor cells in splenocytes collected at the time of sacrifice. (**B**,**E**–**G**) Statistical significance was calculated by *t*-test (**B**,**E**,**G**) or Log-rank (Mantel-Cox) test (**F**) and expressed as * = *p*-value < 0.05; *** = *p*-value < 0.001. Color codes: mice treated with vehicle (black) or R-405 in combination with anti-PD-1 antibody (blue).

In the next series of experiments, we asked whether systemically administered ReHVs can populate a subcutaneously implanted tumor and exert anticancer efficacy. We used a tumor syngeneic with C57BL/6 mice. The model consisted of the ReHV retargeted to the human prostate-specific membrane antigen (hPSMA), named R-405, and the hPSMA-positive LLC1 (LLC1-hPSMA) tumor cells. R-405 exerts anticancer efficacy when administered i.t. [37]. Here, R-405 was administered IV, starting 9 days after tumor implantation, with two injections in combination with the α-PD-1 antibody (schedule in Figure 3A). This combination therapy was chosen because LLC1 tumors are sensitive to co-administration of ReHVs and α-PD-1 [22,30,37,38]. The tumors excised from a subset of mice sacrificed 3 days after the first administration of R-405 showed that the virus was indeed replicating intratumorally, as quantified by the expression of the late viral gene—glycoprotein C (gC); this documents the ability of R-405 to populate the tumor and to replicate (Figure 3B). Of note, gC expression was not detected in the liver, documenting the R-405 specificity (Figure 3B). Figure 3C,D shows that systemically administered R-405 exerted potent anticancer activity against the subcutaneous LLC1-hPSMA tumors, with complete response (CR) in 4 out of 7, partial response (PR) in 2 out of 7, and no response in 1 out of 7 mice. The decrease in tumor growth at day 19 was statistically significant (Figure 3E). The survival curve indicates that 60% of the mice survived (Figure 3F). The mice developed a T cell response towards the tumor, detected as splenocyte reactivity to LLC1-hPSMA as well as to LLC1-wt cells (Figure 3G). Altogether, the IV-administered R-405 was capable of populating the subcutaneous tumor in sufficient amounts to exert anticancer activity and elicit a T-cell response.

### 3.4. Clearance and Biodistribution of Systemically Administered R-337

**Figure 4:**
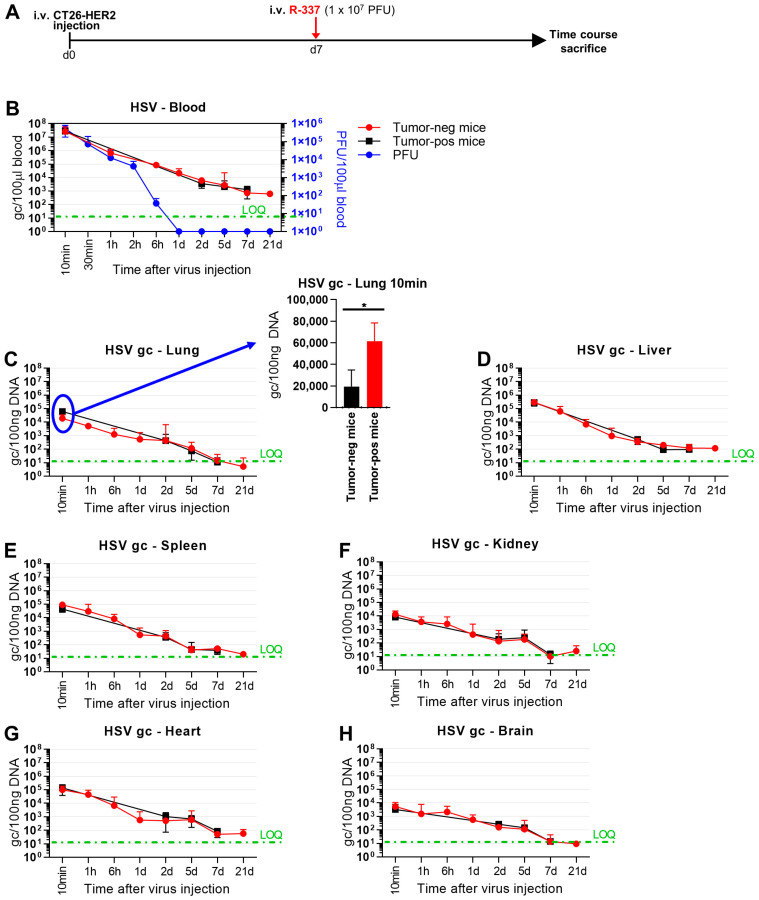

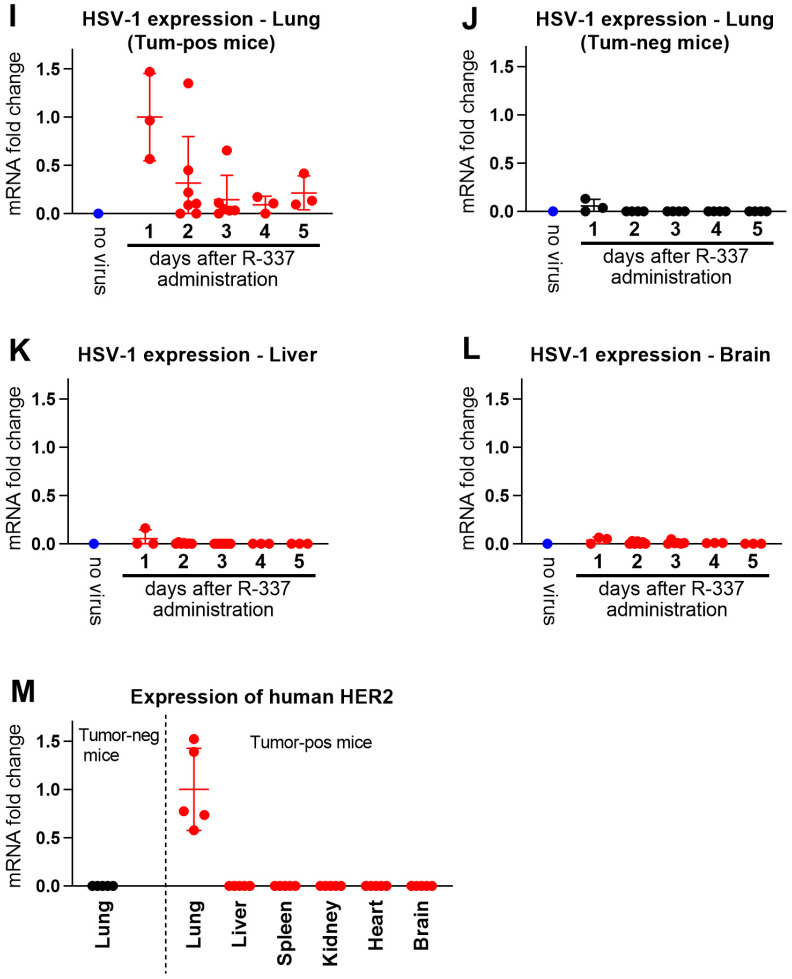
Clearance and biodistribution of systemically administered R-337. (**A**) Treatment scheme. BALB/c-TG mice were injected IV with 1 × 10^6^ CT26-HER2 cells to establish metastatic lung tumors (tumor-positive mice) or PBS (tumor-negative mice). 7 days later, both groups of mice received IV injections of R-337 (1 × 10^7^ PFU) and were sacrificed after 10 and 30 min (both groups), 1, 6, and 24 h (tumor-negative mice only), 2, 5, and 7 days (both groups) and 21 days (tumor-negative mice only). At the time of sacrifice, blood and the indicated tissues were collected, homogenized, and DNA was purified. (**B**) Kinetic of R-337 biodistribution in the blood of tumor-positive and tumor-negative mice, measured as g.c and PFUs. Genome copies were quantified by qRT-PCR on the purified DNAs using a probe specific for HSV DNA polymerase and a standard curve to interpolate the results. Data were expressed as gc/100 µL of blood. The limit of quantification (LOQ, green dashed line) was estimated on the standard curve. Clearance of infectious virus in the bloodstream of tumor-negative mice was determined by titration of blood withdrawn from animals on SK-OV-3 cells and expressed as PFU/100 µL blood. (**C**–**H**) Kinetic of R-337 biodistribution (g.c.) in the lung (**C**), liver (**D**), spleen (**E**), kidney (**F**), heart (**G**), and brain (**H**) of tumor-positive and tumor-negative mice. See panel B for the details. (**I**) Replication of R-337 in the lungs of tumor-positive mice. mRNA levels of viral glycoprotein C were determined by qRT-PCR in lung samples of tumor-positive mice sacrificed 1, 2, 3, 4, or 5 days after IV injection of R-337. Normalized data on endogenous control Rpl13a were reported by setting the mean value of gC expression in R-337 mice sacrificed at day 1 to 1. (**J**) Replication of R-337 in the lungs of tumor-negative mice. (**K**,**L**) Replication of R-337 in the liver (**K**) and brain (**L**) of metastatic lung tumor-positive mice. (**M**) Detection of CT26-HER2 tumors in the indicated organs. Human HER2 was quantified by qRT-PCR on purified DNAs. Data were reported by setting the mean value of HER2 in the lungs of tumor-positive mice to 1. (**B**–**H**) Circles and squares indicate the mean values of 4–5 mice and vertical bars ± SD. (**I**–**M**) Each circle corresponds to an individual mouse; the horizontal line indicates the mean value, and the vertical bars ± SD. (**C**) Statistical significance was calculated by t-test and expressed as * = *p*-value < 0.05. Color code: tumor-positive (red) and negative (black) mice treated with R-337. Untreated mice (blue).

To provide insight into the mechanisms that affect the anticancer efficacy of systemically administered ReHVs, we conducted kinetic clearance and biodistribution analyses of R-337 in the metastatic CT26-HER2 lung model in both tumor-free and tumor-positive mice. Mice were administered IV 1 × 10^7^ PFUs, corresponding to 5.6 × 10^9^ genome copies (g.c.) (Schedule in Figure 4A). Virus clearance from blood was not particularly fast; the amount of circulating virus quantified as g.c. decreased by about 2 logs in 1 h (Figure 4B). Circulating infectious viruses (measured as PFUs) decreased in a similar manner in the first 2 h and by more than 5 orders of magnitude at 6 h. It disappeared completely at 24 h. The finding that the kinetic of virus clearance measured as g.c. was slower than that detected as PFUs was not unexpected since the g.c. determination is carried out by qPCR and detects both intact genomes and genomic fragments as small as 90 bp, and thus also accounts for the degraded virus.

With respect to the tissue biodistribution measured as g.c., R-337 was detected in all tested organs: lung, liver, spleen, kidney, heart, and brain. Whereas the kinetics of disappearance could not be differentiated between tumor-positive and tumor-negative mice in the 1 h–7 d interval (Figure 4C–H), a striking difference was noted very early. Indeed, at 10 min, R-337 g.c. was significantly higher in tumor-positive lungs than in tumor-negative lungs (Figure 4C), indicating that a portion of the virus was indeed captured by the tumor tissue.

To detect replicating viruses, we quantified late viral glycoprotein C (gC) mRNA by qRT-PCR. Figure 4I shows that gC mRNA was present in the lungs of tumor-bearing mice, with the highest levels on day 1 and decreasing levels on subsequent days. Importantly with respect to specificity and safety, gC mRNA was not detected in the lungs of tumor-negative mice, nor in the liver or brain of mice positive for lung metastases (Figure 4J–L). Accordingly, tumor detection by means of HER2 determination confirmed that the lungs of virus-treated CT26-HER2-positive mice were the only organs harboring significant amounts of tumor nodules (Figure 4M). These findings document that viral replication occurred specifically in the tumor-bearing lungs and not in any other analyzed organ.

### 3.5. Lack of Efficacy of Systemically Administered R-337 in HSV-Preimmune Mice

**Figure 5:**
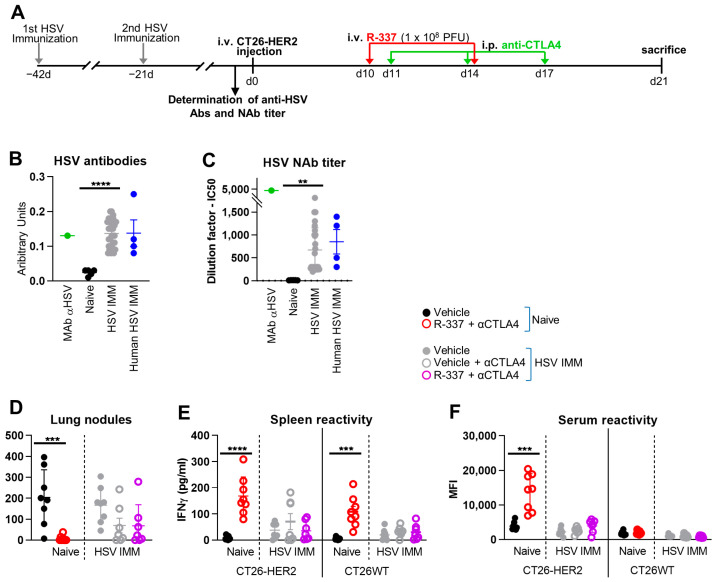
Efficacy of IV therapy with R-337 in combination with anti-CTLA-4 antibody (αCTLA4) on the growth of metastatic CT26-HER2 tumors in HSV-1-immune and naïve mice. (**A**) Late treatment scheme with multiple virus injections. BALB/c-TG mice were immunized twice with HSV-1 virions (**F**) 6 and 3 weeks before tumor cell injection. Immunized and naïve mice were injected IV with 3 × 10^5^ CT26-HER2 cells to establish metastatic lung tumors, and blood samples were taken to verify HSV-1 immunization. 10 days later, mice received 2 IV injections of R-337 (1 × 10^8^ PFUs per injection) every 4 days and 3 i.p. injections of αCTLA4 at 3-day intervals. Mice were sacrificed on day 21 after tumor cell injection. At the time of sacrifice, lungs were harvested, perfused with the staining solution, fixed in Fekete’s solution, and lung metastatic nodules were counted under a microscope. (**B**) Determination of immunization of mice to HSV-1. Sera obtained from the blood of mice (immunized and HSV-1-naïve), HSV-1-positive human patients and anti-HSV-1 MAb HD1 were diluted and reacted with RS cells previously infected with HSV-1, followed by incubation with anti-mouse peroxidase. The association of HSV-1-specific antibodies on the surface of infected cells was measured by the peroxidase reaction. (**C**) Determination of IC50 values of neutralizing antibodies in sera from mice (immunized and HSV-1 naïve), HSV-1-positive human patients, and the anti-HSV-1 MAb HD1. Sera and HD1 were serially diluted and used to neutralize infection with R-8102, an HSV-1-derived virus expressing the β-galactosidase reporter gene. The extent of infection in untreated (control) and serum-treated samples was determined by measuring the β-galactosidase activity. The curve of inhibition of infection versus dilution was used to calculate IC50 values. (**D**) The number of tumor nodules counted on the surface of the lungs. (**E**,**F**) Immune response to CT26-wt and CT26-HER2 tumor cells in splenocytes (**E**) and sera (**F**) collected at the time of sacrifice. Each circle corresponds to an individual mouse; the horizontal line indicates the mean value, and the vertical bars ± SD. (**B**–**D**) Statistical significance was calculated by t-test and expressed as ** = *p*-value < 0.01; *** = *p*-value < 0.001; **** = *p*-value < 0.0001. Color codes: naïve mice treated with vehicle (black) or R-337 + αCTLA4 (red empty circle); HSV-1-immunized mice treated with vehicle (gray), αCTLA4 (gray empty circle), or R-337 + αCTLA4 combination (purple empty circle); HD1 (green); human sera (blue).

Two major obstacles to the success of systemically delivered oncovirotherapy in humans are that the therapy is usually administered to patients carrying advanced tumors, often to patients in whom first-line therapies had failed, and, secondly, that humans bear immunity to the virus. This applies to viruses that are seroprevalent in the population, including the HSV-based ones, as well as to viruses of animal origin that are administered repeatedly and therefore induce seroconversion [23]. To mimic such conditions, we administered the R-337 and α-CTLA4 combination to mice that were HSV-naïve or preimmunized to HSV (HSV-IMM) (schematic schedule, Figure 5A). For anti-HSV immunization, mice received two i.p. injections of purified HSV-1 (F) preparations consisting of a mixture of UV-inactivated and replication-competent viruses. The antibody titer, assessed by CELISA, and the neutralizing antibody (NAb) titer was in the range of the anti-HSV titers seen in human sera (Figure 5B,C). Of note, R-337 was administered 10 days after tumor implantation (i.e., later than in previous experiments) when tumors were well established (Figure 5A). Figure 5D shows that in HSV-naïve mice, R-337 was highly effective and completely protected 100% of the mice. In contrast, in the HSV-IMM mice, the combination of R-337 and α-CTLA4 could not be differentiated from the α-CTLA4 monotherapy (Figure 5D). The T and B cell responses were high in the HSV-naïve arm treated with R-337 and very low in the HSV-IMM arm against both CT26-HER2 and CT26-wt cells (Figure 5E,F). Thus, systemic administration of R-337 to HSV-preimmunized mice failed to produce anticancer protection.

### 3.6. Clearance and Biodistribution of Systemically Administered R-337 in HSV-IMM Mice

**Figure 6:**
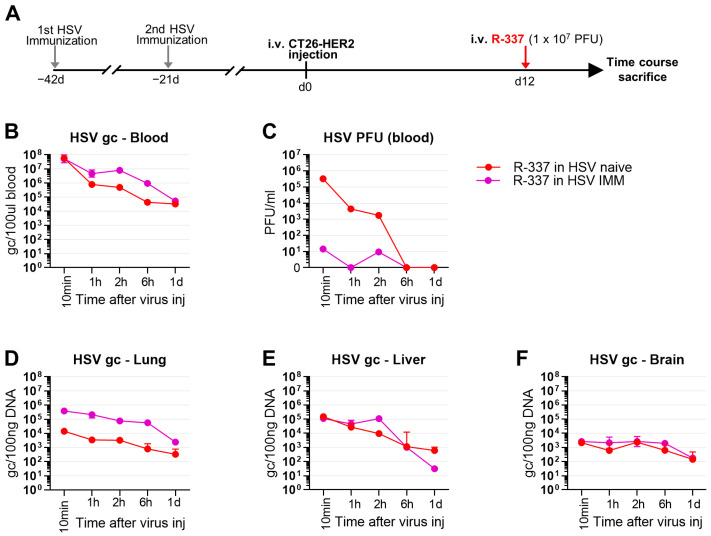
Kinetic of R-337 biodistribution after IV injection in HSV-1-immune and naïve mice. (**A**) Treatment scheme. BALB/c-TG mice were immunized twice with HSV-1 virions (**F**) 6 and 3 weeks before tumor cell injection. Immunized and naïve mice were injected IV with 1 × 10^6^ CT26-HER2 cells to establish metastatic lung tumors and, 12 days later, received IV injections of R-337 (1 × 10^7^ PFU). Subgroups of mice were sacrificed after 10 min, 1, 2, 6 h, and 1 day. At the time of sacrifice, blood and the indicated tissues were collected, homogenized, and DNA was purified. (**B**) Kinetic of R-337 biodistribution (g.c.) in HSV-1-immunized and HSV-1-naïve mice. See Figure 4 for details (**C**) Clearance of infectious R-337 virus in the bloodstream of mice immunized and naïve to HSV-1. See Figure 4 for details. (**D**–**F**) Kinetic of R-337 biodistribution (g.c.) in the lung (**D**), liver (**E**), and brain (**F**) of HSV-1-immunized and HSV-1-naïve mice. Circles indicate the mean values of 4–5 mice and vertical bars ± SD. Color code: mice immunized with HSV-1 (purple) and naïve (red).

To shed light on the factors that hamper ReHV therapy in HSV-IMM mice, we compared the kinetics of blood clearance and biodistribution of R-337 in naïve and HSV-IMM mice at 1, 2, 6, and 24 h after virus administration (schematic schedule in Figure 6A). Blood clearance measured as g.c. was somewhat slower in HSV-IMM mice (Figure 6B). The amount of viral g.c. was higher in the lungs but not in the liver and brain of HSV-IMM mice (Figure 6D–F), hinting that virions in complex with virus-specific antibodies are cleared less rapidly from the blood and possibly from lungs or tend to accumulate in the lungs.

A most remarkable difference between naïve and HSV-IMM mice was seen in the clearance of infectious virus (PFUs) from blood, which was immediate in HSV-IMM mice and took about 6 h in naïve mice (Figure 6C). This finding argues for a very rapid inactivation of R-337 infectivity (10 min) by blood components.

### 3.7. Factors Affecting the Stability of R-337 in Serum and in Whole Blood In Vivo

**Figure 7:**
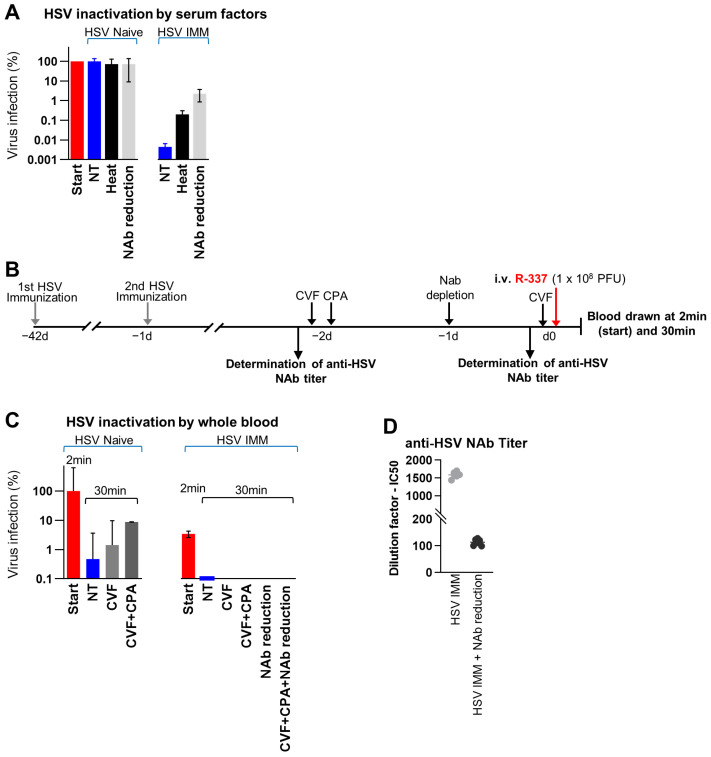
Stability of R-337 in serum and in whole blood in vivo. (**A**) In vitro determination of the ability of HSV-1-immune and naïve sera to inactivate R-337 virions. Sera from the two types of mice were either heated to inactivate complement or treated with Sepharose Protein G to adsorb and reduce the antibodies, including NAbs. Treated and untreated sera were incubated with a fixed amount of R-337 for 30 min, and the amount of infectious virus was determined by titration on SK-OV-3 cells. Results are reported as the percentage of infectious viruses relative to the amount of PFU employed. (**B**) In vivo treatment scheme. BALB/c-TG mice were immunized twice with HSV-1 virions 6 and 3 weeks before R-337 injection. Naïve and HSV-IMM mice were treated twice with Cobra Venom Factor (CVF), 2 days and 1 h before R-337 injection. Other groups of immunized and naïve mice received the same treatment with CVF and the additional administration of cyclophosphamide (CPA) 2 days before the R-337 injection. Other mice were injected with UV-inactivated HSV-1 (NAb reduction) 1 day prior to virus injection. Another group of mice received CVF, CPA, and NAb reduction. After administration of R-337, blood samples were withdrawn after 2 and 30 min. (**C**) Clearance of R-337 PFUs in the bloodstream of HSV-1-immunized and naïve mice subjected to the different treatments or untreated. See Figure 4B for details. At the 2-min time point, the amount of infectious virus in the bloodstream of naïve mice was similar irrespectively from the treatment. Hence its values were averaged to constitute the “start” point. The same approach was applied to the HSV-IMM group. Results are reported as the percentage of infectious viruses relative to the amount of PFU employed. (**D**) Determination of IC50 values of neutralizing antibodies in sera of HSV-1-immunized mice that received the UV-inactivated HSV-1 (NAb reduction). See Figure 5C for details. (**A**,**C**) Each bar indicates the mean value of 3 mice (or the corresponding sera) and vertical bars ± SD.

The main blood components that reduce the half-life of circulating infectious viruses are neutralizing antibodies, complement that acts through both antibody-dependent and antibody-independent pathways, and intrinsic and innate immune responses mediated by soluble factors and cells. We investigated the stability of R-337 in serum and how this is affected by NAbs or by complement in vitro. Replicate aliquots of R-337 virus were incubated with sera from naive or HSV-IMM mice, and the surviving infectious virus was titrated; NAbs were removed by absorption to excess protein G beads; complement was inactivated by heating. It can be seen that naïve sera did not affect virion infectivity, and heat inactivation or antibody depletion left the number of residual PFUs unmodified (Figure 7A). In contrast, HSV-IMM sera inactivated virion infectivity, confirming and extending the results seen in Figure 6C. Depletion of antibodies or heat inactivation substantially, but not completely, rescued infectivity (Figure 7A), hinting that the inactivation induced by HSV-IMM sera was caused mainly by NAbs and in part by complement in the presence of Abs.

For a preliminary assessment of factors that affect the stability of ReHVs in whole blood in vivo, mice were treated as outlined in Figure 7B. Briefly, mice were pre-immunized or not to HSV, then treated with cobra venom factor (CVF) to inactivate complement, with cyclophosphasmide (CPA), a suppressor of some innate responses that increases the amount of oHSV at the tumor site in glioma and sarcoma models [46,47] plus CVF, or partially depleted of NAbs by systemic injections of HSV virions. Finally, R-337 was administered IV, and blood was withdrawn at 2- and 30-min. Figure 7D shows that, despite the fact that the Ab removal resulted in a strong decrease in NAb titer, residual NAbs still exhibited virus neutralization activity (50% inhibition of plaque formation by sera diluted 1:100). The results of the experiment in Figure 7C show that in naïve mice the amount of infectious virus was reduced to 0.5% (2 logs) in 30 min; the decrease was in part reversed by CVF and even more by the CPA-CVF combination. In HSV-IMM mice, the infectivity of R-337 has decreased already at the “start” (2 min after IV injection of virus) and was null (i.e., under the limit of detection) at 30 min, in agreement with the results shown in Figure 6C. CVF and its combination with CPA did not rescue the infectivity of R-337. Partial removal of NAbs was also ineffective in rescuing R-337 infectivity, likely because of the blocking activity exerted by residual NAbs. Partial removal of NAbs also failed to rescue the infection when combined with CVF and CPA. Altogether the results suggest that the in vivo inactivation of R-337 in HSV-IMM mice was largely dependent on NAbs.

## 4. Discussion

This study focused on the systemic delivery of ReHVs and on the conditions that affect the anticancer efficacy, blood clearance, and biodistribution of IV-administered ReHVs.

The intravenously delivered HER2-retargeted onco-immunotherapeutic HSV, named R-337, was found to be effective in inhibiting the growth of lung tumors, generated upon the IV administration of tumor cells, considered a model of metastatic disease [22]. In the current model, the tumor cells were CT26-HER2, syngeneic with the BALB/c mice. To better mimic the situation in humans who develop HER2 tolerance when affected by HER2-positive cancers, the mice were transgenic/tolerant to human HER2 [30]. As monotherapy, a single 1 × 10^8^ PFU dose of R-337 completely abolished tumor growth when administered at early times (Figure 1H). The lower dose of 1 × 10^7^ PFUs still reduced the number of lung nodules and was much more effective when administered in combination with anti-CTLA4 (Figure 2B). Combination therapy also dramatically reduced the number of lung tumors when the virus was administered to mice bearing well-established tumors (Figure 5D, see the naïve mice), i.e., 10 days after tumor implantation, extending previous findings [22]. Systemic administration of ReHV was also effective against subcutaneously implanted tumors (Figure 3). The latter model consisted of R-405, a ReHV retargeted to the human prostate-specific membrane antigen (hPSMA), and LLC1 cells expressing hPSMA that were implanted subcutaneously [37]. R-405 was capable of specifically populating the tumor (Figure 3B). In combination with anti-PD1, it fully protected 60% of the mice (Figure 3F). Systemic T-cell response developed specifically in the virus-treated animals (both monotherapy and combinations) against the TAA (HER2 or hPSMA) and tumor antigens (Figure 2C and Figure 3G). The B-cell response resulted mostly in HER2 and, to a lesser extent, in the tumor cells (Figure 2D). All in all, systemically administered ReHVs in naïve mice exerted a high degree of protection against both the lung metastatic-like and subcutaneous tumors, as monotherapy and in combination therapy.

Mice immunized against HSV (HSV-IMM) carried anti-HSV Abs at a CELISA titer similar to that seen in pooled human sera (Figure 5B,C). In contrast to the results in naïve mice, systemically administered R-337 did not exert anticancer efficacy in HSV-IMM mice, even as combination therapy (Figure 5D). The treatment did not elicit a detectable T cell response to HER2-positive cancer cells nor to the agnostic antigens of the wt tumor cells (Figure 5E). Even the Ab response to the TAA and tumor antigens was almost undetectable (Figure 5F). Current results differ from a previous study of systemically administered ReHV named R-123 [22]. In that study, R-123 was administered in 5 doses every 2–3 days. Treatment started very early, at 3 days after tumor implantation. In retrospect, we interpret that the first doses of the virus actually resulted in a reduction in NAbs (as demonstrated in the last section of the current paper), and that the later injections, at 7 and 9 days, were probably the most effective ones. Effective depletion of NAbs is expected to greatly improve the efficacy of systemically administered ReHVs in HSV-IMM mice and, by extension, in HSV-seropositive patients.

Blood clearance of R-337 in naïve mice, both tumor-negative and lung tumor-positive mice, showed that the virus, measured as g.c., decreased by 3–4 logs in 24 h (Figure 4B). The disappearance of infectious viruses from blood was much faster than that of viral genomes (Figure 4B, compare black and red lines with blue lines). The difference is likely due to the fact that the latter determination detected not only an infectious virus but also a virus in the process of being degraded and that the infectivity of circulating virus was inactivated by blood factors even though the viral genomes, or fragments thereof, were still molecularly detectable. Regarding biodistribution to organs, viral g.c. was found in all organs tested—lung, liver, spleen, kidney, heart, and brain—and decreased over time, between 2 and 5 days (Figure 4). Biodistribution did not differ substantially between tumor-positive and tumor-negative mice. The only remarkable difference was the presence of more virus in the lungs of tumor-positive mice than in the lungs of tumor-negative mice at a very early time after IV administration of ReHV (Figure 4C). This finding suggests that the virus was indeed captured more efficaciously by the tumor. Importantly, ReHV replication, detected as mRNA of the glycoprotein C (gC), a late viral gene, was found exclusively in the tumor-positive lungs and not in any other organs (Figure 4I–L)—i.e., tumor-negative lungs or liver and brain in mice with tumor-positive lungs—confirming and extending previous findings that the R-337 ReHV exclusively replicated in HER2-positive tumor cells.

Blood clearance of R-337 in HSV-IMM mice showed that the circulating infectious virus decreased dramatically in about 10 min, whereas it persisted in the blood of naïve mice for 2–6 h (Figure 6C). In contrast to the infectious virus, viral genome copies were higher in the blood and lungs of HSV-IMM mice, probably as a result of the formation of virus-antibody complexes that were eliminated more slowly than free virus, the prevalent specie in the blood of naïve mice (Figure 6B,D). The rapid disappearance of infectious viruses from the blood of HSV-IMM mice argued for virus inactivation by blood factors. In vitro serum stability and in vivo whole blood stability assays concordantly pointed to neutralizing Abs as the major factor responsible for the decrease (Figure 7).

Earlier studies in nude mice on the anticancer efficacy of OVs in general, including ReHVs, [19,24,41,48,49,50,51,52,53] underscored that the anticancer efficacy was in part due to direct virus-induced oncolysis of the cancer cells. Later investigations in immunocompetent mice highlighted the contribution of the anticancer innate and adaptive responses, as documented by immune cell depletion experiments, by increased efficacy upon combination with checkpoint inhibitors, and by trained immunity [22,30,37,38,39,40,54,55,56,57,58]. On the other hand, current and previous findings highlighted the detrimental effect of blood factors in immune mice, at least on the efficacy of systemically delivered ReHVs. Given this complex situation, the clinical outcome could benefit from exploiting the benefits conferred by virus-induced anticancer immunity and avoiding the pitfalls exerted by antiviral immunity, especially by neutralizing antibodies.

## 5. Conclusions

ReHVs are potent anticancer weapons not only when administered directly into the tumor bed [30,37,38,39,40], but also upon systemic administration to mice harboring disseminated lung cancer nodules or subcutaneous tumors. In such settings, ReHVs were able to populate tumors via the bloodstream, replicate within them, and exert potent anti-tumor efficacy. This occurred in mice not trained to fight the virus, that is, in HSV-naïve mice. When the same OV therapy was administered to HSV-preimmune mice, the virus was rapidly inactivated by neutralizing antibodies and additional antiviral factors. As a result, the anticancer effect was lost.

Blood factors, mainly NAbs, complement, and innate effectors are an obstacle, not only to OVs, including Adenovirus, Measles virus, vaccina, HSV, or other OIVs [59,60], but also to several therapeutic treatments, including therapeutic viral vectors (e.g., Adeno-associated virus for gene therapy [61,62]), viral vaccine vectors, therapeutic approaches to autoimmune disease, strategies to favor transplantation of pig tissues, etc. A variety of efforts [63,64,65] are ongoing to tackle this issue; they include nanotechnology to improve the delivery of OVs in immune individuals [60], the possibility to inactivate complement in humans as performed in COVID-19 patients [66], the clinical application of CPA to augment OV efficacy [67,68,69]. Improvements can also be tailored to the virus, e.g., by shielding virion components or by delivering the virus via carrier cells. These efforts, combined with the higher anticancer efficacy of OIVs, including ReHVs, when administered in combination regimens with ICIs, CAR-Ts, or radio- and chemotherapy [70], and further combined with the high specificity of ReHVs upon systemic administration—confirmed herein as the exclusive replication in targeted cancer tissues and absence of off-tumor and off-target replication—give hope that the systemic delivery of OIVs, and particularly of ReHVs, can be much improved in the near future.

## Data Availability

All the data presented in this study are available in the article.
